# Smart sensors, smarter players: The role of real-time monitoring in football training

**DOI:** 10.1371/journal.pone.0333884

**Published:** 2025-10-29

**Authors:** Ben Liang, Sohom Saha, Hanlei Miao, Liang Chen, Marcin Bialas

**Affiliations:** 1 Faculty of Physical Culture, Gdansk University of Physical Education and Sport, Gdansk, Poland; 2 Henan Sport University, Henan Province, China; 3 Department of Sports Psychology, Lakshmibai National Institute of Physical Education, Gwalior, Madhya Pradesh, India; University of Split, CROATIA

## Abstract

**Objectives:**

This study aimed to develop and evaluate a real-time sensor-based monitoring and feedback system for enhancing four core football performance metrics, passing accuracy, sprint speed, agility, and shot power, each defined and quantified using validated wearable sensors and baseline‐referenced improvement thresholds.

**Methods:**

A randomized controlled trial (RCT) was conducted over eight weeks with 30 university-level male football players (aged 21.70 ± 1.28 years) from Zhengzhou University. Participants were randomly assigned to an experimental group (n = 15), which trained using the real-time monitoring system, or a control group (n = 15), which followed traditional training methods. The wearable system integrated accelerometers, gyroscopes, and magnetometers to provide real-time, skill-specific feedback during drills. Performance data were collected weekly and analyzed using repeated measures ANOVA with effect sizes calculated via partial eta squared (η²p).

**Results:**

The results demonstrated statistically and practically significant improvements in the experimental group across all measured parameters. Notably, the effect sizes ranged from large to very large (η²p = .59 to.89), indicating that the improvements were not only statistically reliable but also substantial enough to have meaningful impact on the players’ performance. Passing accuracy increased by 10.21% (F(1,27) = 210.02, p < 0.001, η²p = .88), sprint speed improved by 17.33% (F(1,27) = 92.00, p < 0.001, η²p = .76), agility improved by 10.90% (F(1,27) = 41.75, p < 0.001, η²p = .59), and shot power increased by 10.75% (F(1,27) = 247.32, p < 0.001, η²p = .89). The control group showed negligible or negative changes in all performance metrics. Performance improvements in the experimental group were progressive and sustained across the 8-week training period, with weekly data showing steady gains in passing accuracy, sprint speed, agility, and shot power. No performance regressions or plateaus were observed during the intervention period.

**Conclusion:**

By delivering instantaneous, sensor-validated feedback on precisely defined performance metrics, the system accelerated improvements in both technical and physical skills. These findings support the integration of wearable sensor technology into football training to achieve data-driven, individualized skill development. Future work should explore AI-driven personalization and long-term retention of gains.

## Introduction

The challenges posed by modern football training schedules necessitate the optimization of training stimuli using minimal effective doses to reduce physical strain on players and enabling their participation in sessions and matches with minimal fatigue [1,2]. This involves applying the necessary training stimulus to maintain or enhance athletic performance [3]. Achieving this balance between specific training methods and their effects amid increasingly congested fixtures presents a significant difficulty [4]. Efforts to mitigate the impact of training volume on players emphasize intensity and reducing overall volume [5,6].

Real-time monitoring and feedback systems have become a valuable solution to overcome the drawbacks of traditional training methods. By utilizing sensor technology, these systems track and assess athlete movements, delivering instant, tailored feedback to athletes and coaches alike [7]. The rationale for using real-time monitoring and feedback in soccer training stems from its ability to speed up skill acquisition, minimize mistakes, and enhance performance by providing immediate corrective insights [8]. Several studies have explored the application of sensor-based systems in soccer training. For instance, Camomilla et al. (2018) designed a wearable inertial measurement unit (IMU) system to evaluate the kinematics of soccer players during specific technical actions. Their results highlighted the effectiveness of these systems in delivering precise movement analysis; however, the research was restricted to post-session evaluation rather than offering real-time feedback [9]. Similarly, Rossi et al. (2018) employed a multi-sensor system to assess soccer players’ performance in small-sided games, shedding light on both physical and tactical elements of gameplay. Nonetheless, their system lacked the capability to deliver real-time feedback to players during training sessions [10]. While technologies such as GPS trackers and inertial measurement units (IMUs) have been widely used to assess workload and general physical performance, these systems typically offer post-session feedback and do not support real-time, task-specific skill correction. The current study introduces a wearable sensor-based system capable of delivering real-time, drill-specific feedback on football skills such as passing accuracy, sprint speed, agility, and shot power, bridging the gap between data collection and in-the-moment skill refinement. This represents an advancement in ecological validity and performance coaching, aligning with the current push toward athlete-responsive, adaptive training systems.

While these studies have made significant contributions to the field, there remain notable gaps in the literature. Firstly, most existing systems focus on physical performance metrics or broad movement patterns, with limited attention to specific technical skills crucial in soccer. Secondly, the integration of real-time feedback mechanisms, particularly for individual skill enhancement, has been underexplored. Lastly, there is a scarcity of comprehensive evaluations of the effectiveness of such systems in improving players’ skills over time. The current study aims to address these gaps by developing and evaluating a novel sensor-based system for real-time monitoring and feedback in soccer training. Our approach focuses specifically on enhancing individual technical skills, such as ball control, passing accuracy, and shooting technique. These four performance variables were selected as they represent core determinants of football performance, frequently cited in both skill assessment literature and coaching framework. They capture a balance of technical execution (passing, shooting) and physical performance (sprint speed, agility), and are considered highly sensitive to targeted training interventions. The main targets of this research are: To design a non-intrusive, wearable sensor system capable of capturing fine-grained data on soccer-specific movements and actions. To develop algorithms for real-time analysis of player performance, with a focus on key technical skills. To implement an immediate feedback mechanism that provides players with actionable insights during training sessions. To evaluate the effectiveness of the system in improving players’ technical skills through a longitudinal study. By addressing these objectives, our study seeks to contribute to the growing body of knowledge on technology-enhanced sports training and provide practical tools for soccer coaches and players to accelerate skill development. The novelty of our approach lies in its focus on real-time, skill-specific feedback for soccer players, potentially revolutionizing how technical training is conducted in the sport.

## Methods

### Study design and participants

This study employed a rigorous randomized controlled trial (RCT) design to assess the effectiveness of a real-time monitoring and feedback system on football skill enhancement. A total of 30 professional male football players, each with a minimum of five years of university-level competitive experience, were recruited from Zhengzhou University School of Physical Education, China. The experimental group underwent an 8-week training intervention that integrated the real-time monitoring and feedback system into their regular training regimen. The control group participated in the same skill assessment protocol as the experimental group, with weekly measurements of passing accuracy, sprint speed, agility, and shot power using the same sensor system and standardized procedures. However, they did not receive any real-time feedback or sensor-based intervention during training. Participants were recruited between (Start date: 23/05/2024) and (End date: 18/07/2024). Informed written consent was obtained from all participants prior to their involvement in the study. Participants were provided with detailed information about the study’s aims, procedures, potential risks, and benefits. Anthropometric variables for experimental group (age: 21.73 ± 1.33 years; weight: 63.66 ± 2.41 kg; height: 1.66 ± 0.03 m; BMI: 22.94 ± 1.20) and control group anthropometric variables (age: 21.66 ± 1.23 years; weight: 63.13 ± 1.84 kg; height: 1.67 ± 0.02 m; BMI: 22.46 ± 0.91) were recorded prior to the intervention. While all participants were professional university-level football players with a minimum of five years of competitive experience, the sample was limited to male athletes aged 19–23 from a single institution (Zhengzhou University). This homogeneity in age and competitive background helped reduce within-group variability, but may also limit the generalizability of the results to broader populations such as younger athletes, female players, or individuals with different training histories. Due to logistical constraints and the availability of athletes, convenience sampling was used within each playing position, which may introduce selection bias. Although baseline characteristics (age, BMI, height, weight) were statistically comparable across groups, other factors such as training history and individual motivation were not directly controlled. Given the demographic and physiological homogeneity of the participants, all being male university-level players within a narrow age (19–23 years) and BMI range, the influence of anthropometric variation on performance outcomes was considered minimal. Consequently, these variables were not included as covariates in the statistical model. However, we acknowledge that in studies involving more heterogeneous samples, controlling for such variables would be more critical. Future studies with larger and more diverse populations should incorporate covariate analyses to further elucidate potential moderating effects. The participants followed regular Chinese Diet. The sample size was determined using G*Power (version 3.1.9.2, University of Kiel, Kiel, Germany) analysis, which indicated that a minimum of 47 participants were required to achieve adequate statistical power (power = 0.95, α = 0.05, effect size = 0.80) [11]. Although the sample size of 30 participants did not meet the projected minimum (n = 47) for a power of 0.95 (α = 0.05, effect size = 0.80) as per G*Power calculations, it reflects a realistic recruitment ceiling for trained, university-level football players available at the study site within the project timeline. To mitigate statistical risk, we applied a repeated-measures design, which increases statistical power by reducing inter-subject variability. Moreover, very high intraclass correlation coefficients (ICC > 0.95) and large observed effect sizes (η²p = .59 to.89) indicate consistent and substantial differences between conditions, supporting the robustness of the findings despite the smaller sample size. Future studies with expanded cohorts should further validate these outcomes. Confidentiality and anonymity were maintained throughout the research process, in compliance with the Declaration of Helsinki guidelines [12].

#### Sampling strategy and randomization.

A stratified random sampling method was employed to ensure balanced representation of playing positions (defenders, midfielders, and forwards) across both the experimental and control groups. Initially, players were categorized into strata based on their primary playing position. Within each stratum, players were then randomly assigned to either the experimental group (n = 15) or the control group (n = 15) using a computer-generated randomization sequence created in Microsoft Excel. The randomization procedure was carried out by an independent researcher who was not involved in participant recruitment, data collection, or analysis, thereby ensuring allocation concealment and minimizing selection bias. Participants were blinded to their group allocation during the initial briefing, and outcome assessors were also blinded to group identity to reduce observer bias during data analysis.

#### Sensor selection and placement.

The study utilized advanced wearable sensors, including accelerometers, gyroscopes, and magnetometers, selected for their proven ability to accurately capture data relevant to football skills [13]. These wearable sensors like accelerometers manufactured by Bosch Sensor tec. GmbH, headquartered in Germany, gyroscopes provided by ST Microelectronics, headquartered in Switzerland and Italy and magnetometers Produced by Honeywell International Inc., headquartered in the United States. To ensure reliability: Accelerometers accuracy ±0.1 m/s²; validated against motion capture systems. Gyroscopes angular velocity error ±0.2°/s; compared with high-speed cameras. Magnetometers measurement error ±0.5%; validated via static and dynamic tests. These sensors were strategically placed on various parts of the players’ bodies, such as the lower back, ankles, and wrists, to ensure comprehensive data collection without hindering movement. The placement of these sensors was based on preliminary tests designed to optimize both data accuracy and player comfort. To ensure these specifications translated effectively into on-field performance tracking, a pre-study validation was conducted comparing the sensor data against high-speed video analysis and motion capture references in controlled test environments. This strategic placement ensured that the sensors did not interfere with the players’ natural movements, thereby providing reliable and valid data. Additionally, the sensors were lightweight and ergonomically designed to minimize discomfort and distraction during training sessions. Prior to each training session, all sensors underwent standardized calibration procedures using static posture alignment and baseline signal stabilization, as per the manufacturer’s protocol. Trained research staff ensured consistent sensor placement on anatomical landmarks (e.g., lower back, wrists, ankles) using elastic straps and marker templates to reduce inter-session and inter-player variability. The same hardware units and firmware versions were used across all participants, and signal checks (battery level, sync timing, noise interference) were performed before each drill. These steps ensured high data integrity and repeatability across sessions. Sensors were placed on the lower back (to capture core stability and whole-body acceleration), ankles (to monitor foot movement and agility), and wrists (for upper-body balance and movement coordination). These positions were selected to target key biomechanical indicators associated with football skills like sprinting, directional changes, passing, and shooting. To validate the appropriateness of these placements, a pre-intervention pilot test was conducted with five non-study football players. Athletes performed the full training protocol while wearing the sensors, and were then asked to report any discomfort, restriction of movement, or perceived performance interference. No adverse effects or alterations to movement mechanics were reported. Additionally, researchers observed the sessions and compared performance metrics (e.g., sprint times) with and without sensors to confirm no significant deviation in natural movement. These findings supported the final sensor positioning protocol used during the full trial.

#### Real-time feedback and visualization.

**Fig 1:**
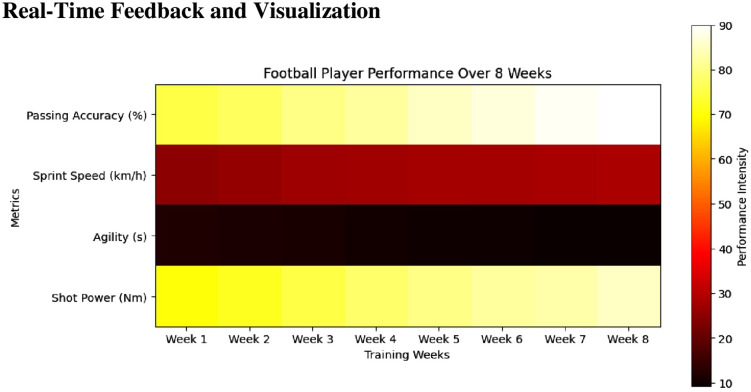
Heatmap analysis of football player performance over 8 weeks: tracking passing accuracy, sprint speed, agility, and shot power. Heatmap visualization of weekly passing accuracy performance for the experimental group over the 8-week training period. The X-axis represents each training week (Week 1 to Week 8), while the Y-axis corresponds to individual players (Player 1 to Player 15). Color intensity reflects the percentage of passing accuracy as recorded by the wearable sensor system, with cooler colors indicating lower accuracy and warmer colors indicating higher accuracy. The heatmap demonstrates a progressive increase in passing accuracy among participants, reflecting consistent skill enhancement as training advanced.

The system provided real-time feedback through intuitive visualizations, including graphs and heatmaps. These visualizations were designed to be user-friendly, enabling players and coaches to quickly interpret the information and make immediate adjustments to training and performance strategies. The feedback mechanism was a critical component of the system, allowing for continuous improvement and fine-tuning of skills. Graphs provided a clear representation of performance trends over time, while heatmaps highlighted areas of high activity and performance intensity. Fig 1 illustrates how passing accuracy improved over time across participants, with the heatmap providing a visual progression from low to high performance zones based on real-time sensor data. The increasing intensity of warm colors across weeks indicates sustained and consistent improvement, reinforcing the quantitative results shown in Table 3. These visualizations were generated using customized MATLAB scripts that processed real-time data from each drill. The processed feedback was displayed on a tablet interface accessible to coaches and players immediately after each session or drill. Heatmaps used color gradients to identify high and low performance zones, e.g., in passing zones or sprint lanes, while trend graphs charted weekly improvements. Although the overall structure of feedback was standardized, performance thresholds were adapted to each player’s baseline, offering semi-individualized cues. Coaches used this data to tailor subsequent training decisions and provide athletes with targeted instructions. This integration of feedback into training not only enhanced engagement but allowed for dynamic and responsive skill correction. This visual feedback was crucial in helping players and coaches identify strengths and areas for improvement, facilitating targeted training interventions.

**Table 3:** Pre- and post-intervention comparison of passing accuracy, sprint speed, agility, and shot power between experimental and control groups.

Variables	Groups	Pre data	Post data	Δ (%)	SS	F	p	η²_p_
Passing Accuracy	EXP	75.67 [3.92]	83.40 [3.38]	10.21	248.31	210.02	*<0.001*	.88
	CG	75.08 [6.48]	74.71 [6.15]	−0.49				
Sprint Speed	EXP	24.05 [1.54]	28.22 [1.25]	17.33	73.01	92.00	*<0.001*	.76
	CG	25.35 [1.76]	25.10 [1.55]	−0.98				
Agility	EXP	12.10 [1.02]	10.78 [.99]	−10.90	5.32	41.75	*<0.001*	.59
	CG	11.81 [.77]	11.69 [.79]	−1.01				
Shot Power	EXP	69.45 [2.22]	76.92 [2.62]	10.75	215.00	247.32	*<0.001*	.89
	CG	70.46 [5.43]	70.36 [5.27]	−0.14				

*EXP: Experimental; CG: Control.

#### Experimental protocol.

A rigorous experimental protocol Table 1 involving professional soccer players. The thirty recruited players were randomly divided into two groups: an experimental group and a control group. The experimental group incorporated the real-time monitoring system into their training regimen, while the control group maintained their regular training practices. Various drills were meticulously designed to assess specific skills, including passing accuracy, sprint speed, agility, and shot power. Passing accuracy was assessed using a standardized target-based scoring system, where players attempted to hit designated target zones and measured through magnetometers [14], sprint speed was measured as the fastest 20m sprint time from a standing start and agility was evaluated using a T-drill test with sensor-based time tracking, both has been measured through accelerometers [15], and shot power was assessed using gyroscopic velocity readings converted to ball speed (m/s) [16]. These four-performance metrics, passing accuracy, sprint speed, agility, and shot power, were selected as “key football skills” based on their established relevance to match performance and skill development in professional players. Each skill was operationally defined and quantitatively measured using validated sensor-based techniques. Passing accuracy was quantified as the percentage of successful target hits using magnetometer data; sprint speed was determined from the best 20-meter sprint time via accelerometers; agility was assessed using T-drill completion time tracked with motion sensors; and shot power was derived from gyroscopic data converted to ball velocity (m/s). Improvement was defined as a statistically significant change from baseline to post-intervention scores, expressed in percentage change and analyzed using repeated measures ANOVA and effect size (η²p) to determine the magnitude of training effects. Each drill was repeated multiple times to ensure the reliability and consistency of the collected data. Performance metrics were gathered during each drill, and real-time feedback was provided exclusively to the experimental group. The drills were standardized to ensure consistency across participants and training sessions, and each session was supervised by trained coaches to maintain protocol adherence. The control group followed traditional training, performing drills without real-time feedback. To assess week-to-week variability, performance was recorded at baseline and at weekly intervals for both groups. This allowed for an analysis of fluctuations in performance trends over time.

**Table 1:** Training protocol for experimental group.

Week	Focus	Warm-Up (15 minutes)	Drills	Cool-Down (10 minutes)
1-2	Foundational Skills	- Jogging (5 minutes) - Dynamic stretches (5 minutes) - Mobility drills (5 minutes)	- Short passing drills (15 minutes) - Agility ladder (10 minutes) - Sprint intervals: 8x20m (30s rest) - Stationary shooting (10 minutes)	- Light jogging (3 minutes) - Static stretching (7 minutes)
3-4	Increased Intensity & Complexity	- Jogging (5 minutes) - Dynamic stretches (5 minutes) - Mobility drills (5 minutes)	- Passing drills with movement (20 minutes) - Agility with directional changes & ball (12 minutes) - Sprint intervals: 10x30m (30s rest) - Shooting with movement into space (15 minutes)	- Light jogging (3 minutes) - Static stretching (7 minutes)
5-6	Speed & Precision under Pressure	- Jogging (5 minutes) - Dynamic stretches (5 minutes) - Mobility drills (5 minutes)	- Passing under pressure (25 minutes) - Agility cone drills (15 minutes) - Sprint intervals: 10x40m (20s rest) - Shooting under defensive pressure (20 minutes)	- Light jogging (3 minutes) - Static stretching (7 minutes)
7-8	Game Realism & High-Intensity	- Jogging (5 minutes) - Dynamic stretches (5 minutes) - Mobility drills (5 minutes)	- Game-realistic passing & small-sided games (30 minutes) - Sprint intervals: 6x60m (10s rest) - Advanced agility with reaction training (15 minutes) - Complex shooting drills under match conditions (25 minutes)	- Light jogging (3 minutes) - Static stretching (7 minutes)

*Frequency: 5 days a week up to 8^th^ week.

*Progression: Training intensity increases as the weeks progress, focusing on more game-realistic situations in the latter weeks.

#### Control group training protocol.

The control group continued with their standard football training routine as prescribed by the university team’s coaching staff. This included general drills for passing, sprinting, agility, and shooting, similar in structure and volume to those used in the experimental group. However, they did not receive any real-time sensor-based feedback, visualizations, or personalized performance data. Training intensity and frequency (5 days per week over 8 weeks) were kept consistent with the experimental group to control for training load. All performance evaluations for the control group, including passing accuracy, sprint speed, agility, and shot power, were conducted using the same sensor setup and measurement procedures, but only during designated test sessions at baseline and at each weekly interval. This ensured consistent and unbiased data collection for both groups while isolating the effect of real-time feedback as the primary intervention variable.

#### Data collection and processing.

Raw sensor data were wirelessly transmitted to a central processing unit and analyzed using a customized MATLAB (R2023a, MathWorks, USA) script. Data processing steps included:

1. **Filtering:** Noise reduction using a 4th-order Butterworth low-pass filter (cutoff frequency: 10 Hz) [17].
2. **Data Fusion:** Combining multiple sensor data streams to enhance measurement accuracy [18].
3. **Feature Extraction:** Identifying relevant performance metrics such as ball contact time, acceleration, and angular velocity.

The intraclass correlation coefficient (ICC) for all performance measures was > 0.95, indicating excellent reliability **Table 2**.

**Table 2:** Interclass correlation coefficient (ICC).

Variables	ICC
Passing Accuracy	.971
Sprint Speed	.961
Agility	.975
Shot Power	.998

### Data analysis

Data were analyzed using SPSS (version 26), with the significance level set at p < 0.05. Descriptive statistics, including mean and standard deviation, were calculated for all variables. A repeated measures analysis of variance (ANOVA) was conducted to examine within-group and between-group differences across time points for the variables: passing accuracy, sprint speed, agility, and shot power. Effect sizes were determined using partial eta squared (η²p) to assess the magnitude of the intervention’s effects. Effect sizes were interpreted according to Cohen’s guidelines: small (η²p ≥ 0.01), medium (η²p ≥ 0.06), and large (η²p ≥ 0.14) [19]. Data normality was assessed through the Shapiro-Wilk test, ensuring the assumptions of ANOVA were met.

### Ethical considerations

This study was conducted prospectively and adhered to established ethical guidelines. Ethical approval was obtained from the Zhengzhou University School of Physical Education Research Committee (Approval Number: IEC/ZZU/SPE/493). Before participation, all individuals provided written informed consent after receiving a comprehensive explanation of the study’s objectives, procedures, potential risks, and benefits. Participants were informed of their right to withdraw at any stage without penalty. Confidentiality was ensured through anonymization of all data, and research materials were securely stored.

## Results

The Table 3 presents the comparative analysis of pre- and post-intervention data for passing accuracy, sprint speed, agility, and shot power between the experimental (EXP) and control (CG) groups. The EXP group exhibited significant improvements across all performance variables, with passing accuracy increasing by 10.21% (p < 0.001, η²p = .88), sprint speed improving by 17.33% (p < 0.001, η²p = .76), agility enhancing by 10.90% (p < 0.001, η²p = .59), and shot power increasing by 10.75% (p < 0.001, η²p = .89). Conversely, the CG group demonstrated negligible or negative changes across all parameters. The statistical analysis, including sum of squares (SS), F-values, and partial eta squared (η²p), confirms the substantial effect of the intervention on the EXP group, reinforcing the efficacy of the applied training methodology in enhancing football performance attributes.

## Discussion

The observed performance improvements can also be interpreted through the lens of motor learning theory, particularly the constraints-led approach (CLA), which emphasizes the dynamic interaction between task, environment, and performer. By delivering real-time augmented feedback, the system functioned as a task constraint that guided attention and reinforced movement corrections, an application consistent with external feedback theories that support skill acquisition through enhanced error detection and correction. These insights suggest that real-time feedback systems could play a meaningful role in shaping motor behavior and accelerating learning curves in sport-specific contexts. The improvements observed in the experimental group attributed to the neuromuscular adaptations, biomechanical efficiency, and sport-specific motor learning principles embedded in the training protocol. Sprint speed, a crucial factor in football performance, was significantly enhanced in the experimental group. The observed improvements in sprint speed and agility are likely underpinned by specific neuromuscular adaptations elicited through repeated exposure to multidirectional movements and explosive sprint drills. Sprint intervals and directional change drills likely enhanced motor unit recruitment and synchronization, contributing to a greater ability to produce force rapidly. Training stimuli such as high-velocity sprints and agility ladders also promote improvements in the rate of force development (RFD), a critical factor for sprint acceleration and change of direction. In agility-based tasks, athletes are required to execute quick deceleration and re-acceleration phases, which condition the eccentric-concentric transition in muscle-tendon units, improving reactive strength and muscle, tendon stiffness. Furthermore, the repeated execution of game-like movement patterns may enhance proprioceptive feedback, intermuscular coordination, and anticipatory timing, all of which contribute to more efficient neuromechanical responses during rapid changes of direction. Over time, these adaptations likely led to improved efficiency in force application, reduced ground contact time, and better stride mechanics, all of which directly contribute to the measurable improvements in sprint speed and agility. The reliability and consistency of the real-time feedback mechanism were ensured through multiple layers of system validation and quality control. The sensors were pre-validated in controlled environments using motion capture and high-speed video comparisons. Additionally, standardized calibration and consistent placement protocols were implemented before each training session. The use of identical hardware and firmware across all units, coupled with pre-drill battery and signal checks, minimized device-related variability. These steps contributed to excellent data integrity, as evidenced by the high intraclass correlation coefficients (ICC > 0.95) across all performance metrics. The visual feedback (graphs and heatmaps) was derived from standardized improvement thresholds based on baseline data, ensuring consistency across sessions and players. This structured and repeatable process enabled the feedback to be both actionable and reliable, supporting its role in enhancing player performance.

This enhancement ascribed to the repetitive execution of explosive movements integrated within skill-based drills [20]. The emphasis on acceleration and deceleration mechanics during dribbling, quick directional changes, and short sprints in game-like scenarios likely contributed to improved neuromuscular coordination and stride efficiency [21]. Furthermore, the dynamic nature of skill-based football drills necessitates frequent recruitment of fast-twitch muscle fibers (Type IIa and Type IIx), thereby augmenting power production and sprinting capacity [22]. The application of sport-specific sprinting tasks also led to improvements in reactive strength, ground contact time reduction, and enhanced rate of force development, all of which play a pivotal role in sprinting performance [23]. Agility, a key component of football that involves rapid changes in direction and body orientation, demonstrated significant improvement post-training. The skill-based football training regimen incorporated multidirectional movement patterns, reactive agility drills, and sport-specific change-of-direction tasks that contributed to neuromuscular adaptation [24]. Enhancements in agility explained through increased proprioceptive awareness, improved muscle-tendon stiffness, and heightened intermuscular coordination, which collectively enhance movement efficiency [25]. The repetitive execution of cutting, pivoting, and lateral movements during small-sided games and skill drills likely facilitated neural adaptations, resulting in reduced decision-making latency and improved reaction time [26]. Additionally, the emphasis on balance, stability, and kinesthetic awareness within the training protocol further contributed to agility enhancement [27]. The significant improvement in passing accuracy observed in the experimental group underscores the efficacy of skill-based football training in refining technical precision and motor control. Passing accuracy in football is largely dependent on a player’s ability to synchronize visual perception, cognitive processing, and precise motor execution [28]. The integration of passing drills in dynamic and variable environments likely led to improvements in visuomotor coordination, spatial awareness, and execution efficiency [21]. Repetitive exposure to controlled passing exercises, combined with time-pressure decision-making tasks, likely enhanced the players’ ability to adjust foot placement, force application, and angular precision during ball striking [29]. Additionally, improvements in lower limb proprioception and kinesthetic sense facilitated superior ball control, allowing for more precise and consistent passes under various game conditions. While proprioception and kinesthetics awareness were not directly measured in this study, their contribution to passing accuracy is well-supported in the motor learning and skill acquisition literature. The real-time feedback system likely enhanced these neurosensory components by promoting immediate movement correction and reinforcing sensorimotor mapping. Specifically, feedback-driven adjustments during repeated passing drills may have improved athletes’ awareness of joint position, limb velocity, and movement timing, key elements of lower limb proprioception. This enhanced body awareness can result in more consistent foot placement, force application, and coordination during ball contact, all of which are critical for passing precision. Future studies should consider integrating proprioceptive assessment tools (e.g., joint position sense tests) to directly quantify these adaptations.

The enhancement in shooting power among the experimental group attributed to the biomechanical and neuromuscular adaptations resulting from skill-based training. Shooting power in football is a function of optimal kinetic chain activation, force generation, and energy transfer through coordinated muscle actions [30]. The repetitive execution of shooting drills under varying intensities and environmental constraints likely facilitated neuromuscular efficiency, enabling players to generate greater force during ball contact. Furthermore, skill-based training that emphasizes explosive kicking movements contributed to increased lower limb strength, enhanced rate of force development, and improved hip flexor and extensor activation [31]. The progressive overload principle applied through repeated maximal-effort shooting attempts fostered strength adaptations in the quadriceps, hamstrings, and gastrocnemius, which are primary contributors to powerful ball striking [32]. Additionally, improvements in postural control and dynamic balance further supported the transfer of kinetic energy from the lower limbs to the ball, ultimately enhancing shooting power. The observed improvements in sprint speed, agility, passing accuracy, and shooting power collectively highlight the effectiveness of skill-based football training in eliciting physiological and neuromuscular adaptations relevant to game performance [21]. The specificity of training, characterized by sport-relevant movement patterns, reactive decision-making, and high-intensity skill execution, likely facilitated superior neuromotor adaptations compared to traditional training modalities [33]. The integration of cognitive, perceptual, and motor skill development within the training paradigm further reinforced the efficiency and transferability of these adaptations to actual match scenarios [34]. These findings align with the principles of motor learning and dynamic systems theory, which suggest that repeated exposure to sport-specific stimuli enhances movement coordination, efficiency, and automaticity [35]. Moreover, the improvements attributed to enhanced sensorimotor integration, increased synaptic plasticity, and greater activation of neural pathways associated with technical execution in football. While the findings revealed statistically significant and practically meaningful improvements across all performance metrics, the relatively small sample size (n = 30) presents a potential risk of overfitting or inflated effect estimates. No cross-validation or bootstrapping procedures were conducted due to the fixed and limited sample of professional players available for this study. However, several methodological strengths, including the use of a randomized controlled design, repeated measures over 8 weeks, very high intraclass correlation coefficients (ICC > 0.95), and large observed effect sizes (η²p ranging from.59 to.89), increase confidence in the robustness and reliability of the results. Future studies with larger and more diverse samples should incorporate cross-validation and sensitivity analyses to confirm these findings and enhance their generalizability.

While the real-time feedback system demonstrated substantial effectiveness in structured training settings, its applicability in real-game scenarios or high-pressure environments may be limited. The system’s current design primarily supports controlled drills, where athletes can process feedback and adjust their technique in real time. However, during competitive match play, athletes often lack the cognitive bandwidth to process external feedback amidst dynamic stimuli and decision-making pressures. Moreover, the current system does not differentiate between nuanced technical errors (e.g., foot placement angle vs. contact timing), which could limit its precision for skill-specific correction. Future iterations should explore context-sensitive feedback, such as haptic or delayed auditory cues, and machine learning integration to offer personalized and adaptive recommendations. Research is also needed to evaluate the system’s effectiveness under match-simulated or fatigue-induced conditions, where real-time processing may be constrained. The findings of this study have direct implications for football coaching and athlete development. Coaches can integrate real-time feedback systems into technical drills to accelerate skill acquisition by providing immediate, objective data on movement execution. This can be particularly useful during small-sided games, individualized training sessions, or rehabilitation protocols, where real-time cues help correct inefficiencies as they occur. However, implementation challenges remain. High costs of sensor equipment, the need for technical training to interpret feedback data, and logistical demands (e.g., calibration, maintenance) may limit widespread adoption, especially in grassroots or resource-constrained environments. To address this, future systems should focus on cost-effective, user-friendly solutions with automated insights that can be used without extensive technical expertise. Collaborations between sports scientists, tech developers, and coaches will be essential to tailor these technologies for real-world integration. Although this study focused on football, the principles of real-time, sensor-driven feedback can be extended to other sports requiring complex motor skill execution, such as basketball, tennis, or hockey. However, scalability may be limited by access to equipment and technical expertise. Future work should explore low-cost adaptations and mobile-based systems to support implementation in grassroots settings. Moreover, long-term studies should investigate whether short-term skill gains persist over time and translate into improved decision-making and performance under pressure.

## Conclusion

This study presents compelling evidence for the efficacy of real-time sensor-based monitoring and feedback systems in enhancing football performance. The significant improvements observed across all measured parameters in the experimental group underscore the value of immediate, data-driven feedback in skill development. The system’s success can be attributed to several key factors: (1) the precision of real-time performance monitoring through strategically placed sensors, (2) the immediate delivery of actionable feedback, and (3) the integration of this technology within a structured training protocol. The marked improvements in passing accuracy (10.21%), sprint speed (17.33%), agility (10.90%), and shot power (10.75%) demonstrate the system’s comprehensive impact on both technical and physical aspects of performance. These enhancements are particularly noteworthy given the professional level of the participants and the relatively short intervention period of eight weeks. The substantial effect sizes (η²p ranging from.59 to.89) further validate the practical significance of these improvements. Our findings suggest that the integration of sensor-based feedback systems can accelerate skill acquisition and refinement in football training. The system’s ability to provide immediate, objective feedback addresses a critical gap in traditional training methods, where feedback is often delayed and subjective. This approach aligns with modern principles of motor learning and skill acquisition, suggesting that immediate, precise feedback can enhance the rate and quality of skill development. However, several limitations should be considered in future research. The sample size, while sufficient for statistical analysis, could be expanded in future studies to enhance generalizability. Additionally, long-term follow-up studies could evaluate the persistence of performance improvements and the system’s effectiveness across different skill levels and age groups. Future research directions should explore the potential applications of this technology in other sports, the integration of artificial intelligence for more sophisticated feedback mechanisms, and the development of more specialized sensor configurations for specific skill development. The positive results of this study suggest that real-time sensor-based feedback systems could become an integral component of modern sports training methodologies. These findings have significant implications for coaches, athletes, and sports scientists, offering a validated approach to enhance training efficiency and effectiveness. The system’s demonstrated success in improving multiple performance parameters suggests its potential value in both individual skill development and team training contexts.

## Supporting information

S1 File Raw data. (XLSX)
